# Clinical efficacy and safety in patients treated with teicoplanin with a target trough concentration of 20 μg/mL using a regimen of 12 mg/kg for five doses within the initial 3 days

**DOI:** 10.1186/s40360-020-00424-3

**Published:** 2020-07-08

**Authors:** Takashi Ueda, Yoshio Takesue, Kazuhiko Nakajima, Kaoru Ichiki, Kaori Ishikawa, Yoshiko Takai, Kumiko Yamada, Toshie Tsuchida, Naruhito Otani, Yoshiko Takahashi, Mika Ishihara, Shingo Takubo, Hiroki Ikeuchi, Motoi Uchino, Takeshi Kimura

**Affiliations:** 1 Department of Infection Control and Prevention, Hyogo College of Medicine, 1-1 Mukogawa-cho, Nishinomiya, Hyogo 663-8501 Japan; 2 Department of Public Health, Hyogo College of Medicine, Nishinomiya, Hyogo Japan; 3 Department of Pharmacy, Hyogo College of Medicine Hospital, Nishinomiya, Hyogo Japan; 4 Department of Inflammatory Bowel Disease, Hyogo College of Medicine, Nishinomiya, Hyogo Japan

**Keywords:** Teicoplanin, Loading dose, Trough concentration, Hypoalbuminemia, Therapeutic drug monitoring

## Abstract

**Background:**

A trough concentration (C_min_) ≥20 μg/mL of teicoplanin is recommended for the treatment of serious methicillin-resistant *Staphylococcus aureus* (MRSA) infections. However, sufficient clinical evidence to support the efficacy of this target C_min_ has not been obtained. Even though the recommended high C_min_ of teicoplanin was associated with better clinical outcome, reaching the target concentration is challenging.

**Methods:**

Pharmacokinetics and adverse events were evaluated in all eligible patients. For clinical efficacy, patients who had bacteremia/complicated MRSA infections were analyzed. The primary endpoint for clinical efficacy was an early clinical response at 72–96 h after the start of therapy. Five dosed of 12 mg/kg or 10 mg/kg was administered as an enhanced or conventional high loading dose regimen, respectively. The C_min_ was obtained at 72 h after the first dose.

**Results:**

Overall, 512 patients were eligible, and 76 patients were analyzed for treatment efficacy. The proportion of patients achieving the target C_min_ range (20–40 μg/mL) by the enhanced regimen was significantly higher than for the conventional regimen (75.2% versus 41.0%, *p* < 0.001). In multivariate analysis, C_min_ ≥ 20 μg/mL was an independent factor for an early clinical response (odds ratio 3.95, 95% confidence interval 1.25–12.53). There was no significant difference in the occurrence of adverse events between patients who did or did not achieve a C_min_ ≥ 20 μg/mL.

**Conclusion:**

A target C_min_ ≥ 20 μg/mL might improve early clinical responses during the treatment of difficult MRSA infections using 12 mg/kg teicoplanin for five doses within the initial 3 days.

## Background

Teicoplanin is a glycopeptide antibiotic used for the treatment of methicillin-resistant *Staphylococcus aureus* (MRSA). Teicoplanin inhibits peptidoglycan polymerization, resulting in the inhibition of bacterial cell wall synthesis and cell death [1]. This antibiotic is currently available in many countries in Europe, Asia, and South America but not in the United States. Approximately 90% of teicoplanin bound to serum albumin and is present at high levels in tissues, which may explain its long half-life (83–168 h). Because steady state is generally achieved in five half-lives, 14 days of repeated administration is required to reach 93% of the concentration at steady state [2]. Therefore, a loading dose of teicoplanin is required to achieve early optimal serum levels [3]. The ratio of the area under the concentration-time curve to the minimum inhibitory concentration (AUC/MIC) was used to determine the pharmacokinetic/pharmacodynamic (PK/PD) index associated with teicoplanin therapy [4]. In a clinical setting, the trough concentration (C_min_) is used as a surrogate marker to predict adequate treatment effects [5]. Although the C_min_ is recommended to be obtained 4 days after the start of therapy, it might be acceptable to perform therapeutic drug monitoring (TDM) within 3 days in cases of low renal function.

Traditionally, a C_min_ ≥ 10 μg/mL is considered appropriate for MRSA infections [6]. Recently, it was reported that a teicoplanin C_min_ ≥ 15 μg/mL was required for the successful clinical treatment of MRSA infection [7, 8], whereas a C_min_ ≥ 20 μg/mL was recommended for other serious infections such as bone and joint infections and infective endocarditis [9, 10]. Wilson et al. [9] showed that treatment in 6/10 staphylococcal infective endocarditis patients failed if the C_min_ was < 20 μg/mL compared with 1/11 where the C_min_ was ≥20 μg/mL. Byrne et al. [11] reported that the mean C_min_ on days 3–7 in successful cases was 19.6 mg/L, suggesting that a target C_min_ ≥ 20 mg/L would be required for the clinically acceptable probability of a successful outcome. However, Harding et al. [12] reported that with the standard dose, most patients had a C_min_ < 15 μg/mL; therefore, they could not conclude that a C_min_ ≥ 20 μg/mL would add further benefit. Byrne et al. [11] reported that although their hospital adopted higher than conventional doses in patients with hematological malignancy with the aim of achieving a C_min_ ≥ 20 μg/mL, attainment of the target concentration in the first week of therapy was poor.

A dosing regimen of teicoplanin to reach a C_min_ ≥ 20 μg/mL should be used for patients with severe, deep-seated or complicated MRSA infections. For bone and joint infections and infective endocarditis, teicoplanin 12 mg/kg body weight every 12 h for three-to-five doses is recommended [6]. In Monte Carlo simulations, a high probability of attaining the target C_min_ of 20 μg/mL was observed using a regimen of 12 mg/kg administered at 12-h intervals for five doses, but not when using four doses [13]. Byrne et al. [14] reported the recommended loading dose to achieve a C_min_ ≥ 20 μg/mL based on population PK analysis was 12 mg/kg administered every 12 h for five doses in patients with a body weight of 70 kg and serum albumin level of 3.0 mg/dL. This enhanced loading dosing regimen was considered optimal on the basis of these simulation analyses. Taken together, sufficient clinical data to support C_min_ ≥ 20 μg/mL have not been obtained. Even though the recommended high C_min_ of teicoplanin appeared to be associated with a better clinical outcome, reaching the target concentration is challenging. Regimens to attain this target concentration have only been suggested by PK/PD analyses. The aim of this study was to evaluate the clinical efficacy and safety when the target teicoplanin C_min_ was set as ≥20 μg/mL in patients with complicated MRSA infections including bacteremia using a loading dose regimen of 12 mg/kg administered every 12 h for five doses.

## Methods

### Patients

This retrospective study was conducted between June 2015 and May 2019, and was approved by the Institutional Review Board of Hyogo College of Medicine (No. 3266). Adult patients who were treated with teicoplanin, and in whom TDM was performed, were included in the study. Exclusion criteria were patients with known hypersensitivity to teicoplanin, pregnancy, below the age of 18 years, and requirement of intermitted hemodialysis and continuous renal replacement therapy. The analysis of C_min_ and the safety population included all eligible patients. The analysis of the clinical efficacy population included patients 1) who had bacteremia or complicated infections [ventilator associated pneumonia (VAP), osteomyelitis and arthritis infection, and central nervous system infection] by MRSA, 2) who received at least 4 days of teicoplanin treatment, 3) who did not receive any concomitant antibiotics with anti-MRSA activity, and 4) who did not receive the above mentioned antibiotics for > 24 h within the previous 3 days.

A diagnosis for each infection was based on definitions in the guidelines issued by the National Healthcare Safety Network [15]. Infections with at least one of the following signs were analyzed: core temperature > 37.8 °C, total peripheral white blood cell (WBC) count > 10,000/mm^3^, or C-reactive protein (CRP) > 3.0 mg/dL. The minimum inhibitory concentration (MIC) of teicoplanin was measured by microdilution methods in accordance with the Clinical and Laboratory Standards Institute testing guidelines (M02 and M07, 2018) [16]. MIC break-points set by the European Committee on Antimicrobial Susceptibility Testing were adopted in this study, and antimicrobial resistance was defined as MIC ≥4 μg/mL. The estimated glomerular filtration rate (eGFR) was calculated using the following formula developed by the Japanese Society of Nephrology [eGFR (mL/min/1.73 m^2^) = 194 × serum creatinine ^(− 1.094)^ × age ^(− 0.287)^ × 0.739 (for females)] [17].

### Administration plan in patients with conventional and enhanced high loading dose regimens

The target initial C_min_ was 15–30 μg/mL between June 2015 and May 2018, and 20–40 μg/mL in patients with bacteremia/complicated MRSA infections between June 2018 and May 2019. In accordance with these target C_min_ values, we conducted two different teicoplanin dose regimens for 3 consecutive days (Table 1). A conventional high loading dose regimen was used for patients with a target C_min_ 15–30 μg/mL, and an enhanced high loading dose regimen was used for patients with a target C_min_ 20–40 μg/mL.

**Table 1:** Teicoplanin dosing regimen according to renal function

Conventional high loading dose regimen						Enhanced high loading dose regimen					
eGFR (ml/min /1.73 m^2^)	The total dose for the initial 3 days				Maintenance dose after day 3	eGFR (ml/min /1.73 m^2^)	The total dose for the initial 3 day				Maintenance dose after day 3
	1st day	2nd day	3rd day	Total			1st day	2nd day	3rd day	Total	
≥60	10 mg/kg twice daily	10 mg/kg twice daily	10 mg/kg once daily	50 mg/kg	6.7 mg/kg once daily	≥60	12 mg/kg twice daily	12 mg/kg twice daily	12 mg/kg once daily	60 mg/kg	6.7 mg/kg once daily
40–60	10 mg/kg twice daily	10 mg/kg once daily	10 mg/kg once daily	40 mg/kg	3.3 mg/kg once daily	30–60	12 mg/kg twice daily	12 mg/kg once daily	12 mg/kg once daily	48 mg/kg	5.0 mg/kg once daily
< 40	10 mg/kg twice daily	6.7 mg/kg once daily	6.7 mg/kg once daily	33.4 mg/kg	5.0 mg/kg every 2 days	< 30	12 mg/kg twice daily	12 mg/kg once daily	6.7 mg/kg once daily	42.7 mg/kg	6.7 mg/kg every 2 days

*eGFR* estimate glomerular filtration rate

Conventional high loading dose regimen for patients with eGFR ≥60 mL/min/1.73 m^2^: a loading dose of 10 mg/kg (actual body weight) twice daily on the first and second days, followed by 10 mg/kg once daily on the third day. Maintenance dosing after the fourth day was 6.7 mg/kg once daily. Loading and maintenance dose was adjusted according to renal function (Table 1). Enhanced high loading dose regimen for patients with eGFR was ≥60 ml/min/1.73 m^2^: a loading dose of 12 mg/kg twice daily on the first and second days, followed by 12 mg/kg once daily on the third day The maintenance dosing regimen after the fourth day was 6.7 mg/kg once daily. Loading and maintenance dose was adjusted according to renal function (Table 1).

### Therapeutic drug monitoring and dosage adjustment

An initial C_min_ sample was obtained prior to the administration of teicoplanin on the fourth day (at 72 h after the first dose). The target C_min_ was defined as 20–40 μg/mL. The dose of teicoplanin was adjusted according to the initial C_min_. Additional loading doses were administered on the fourth day if the initial C_min_ was lower than the target C_min_. Blood samples were collected in blood-collection tubes without a blood coagulation accelerator and immediately centrifuged at 3000 rpm for 10 min. Teicoplanin was measured using a fluorescence polarization immunoassay with a TDXFLX analyzer (Abbott Japan Co., Tokyo, Japan) and a teicoplanin TDM kit-IBL (OXIS International Inc., Beverly Hills, CA, USA).

### Clinical efficacy

The primary endpoint was an early clinical response at 72–96 h after the start of teicoplanin therapy. We defined patients as responders if they had a 30% or greater decrease in total peripheral WBC count or CRP, decline of fever (defined as a daily maximum temperature decrease of > 0.3 °C for at least two consecutive days in febrile patients), without worsening of clinical features, and did not die within 96 h [18]. Secondary efficacy end points were clinical success at the end of teicoplanin therapy (EOT), which was defined as survival with the resolution or improvement of all core symptoms and signs of infection in each infection to the extent that further antibacterial therapy with anti-MRSA activity was unnecessary. Microbiological assessments were conducted using cultures taken before the start of teicoplanin administration and at the completion of treatment, and microbiological success was defined as “eradication” (pathogen absent in culture) or “presumed eradication” (no material available for culture because the infection was cured or attenuated).

### Adverse events

Adverse events of nephrotoxicity and hepatotoxicity were evaluated on the fourth day of therapy and at the end of teicoplanin therapy. Nephrotoxicity was defined as a serum creatinine (Cre) increase > 0.5 mg/L or 50% increase from the baseline [19]. Hepatotoxicity was defined as aspartate aminotransferase (AST) or alanine aminotransferase (ALT) levels at or above three times the upper limit of normal. If the AST or ALT baseline was abnormal, hepatotoxicity was defined as AST or ALT at or above three times the baseline [20].

### Statistical analysis

Parametric variables were analyzed using the Student’s *t*-test, while nonparametric variables were analyzed using the Mann–Whitney *U*-test or Fisher’s exact test. Multivariate analyses were performed to determine the odds ratio (OR) to achieve the target C_min_ (≥20 μg/mL) and early clinical responses. The crude OR in univariate analysis was estimated for each variable using the chi-squared test, and potential confounders were examined by cross tabulation. Variables selected by univariate analysis (*p* < 0.1) were subsequently entered into a stepwise logistic regression model to estimate the magnitude of association [adjusted OR and 95% confidence interval (CI)]. The level of significance was set at *p* < 0.05. SPSS ver. 24 (SPSS Inc., Chicago, IL, USA) was used to perform statistical analyses.

## Results

### Patient characteristics

The number of patients included in the analysis of C_min_ and the safety population was 512 (363 in the high loading dose regimen group and 149 in the enhanced high loading dose regimen group). Among 139 patients with MRSA infections, 63 were excluded from the efficacy population [26 because of the previous use of antimicrobial agents with anti-MRSA activity and 37 without bacteremia/complicated MRSA infections (skin and soft tissue infection = 21; intra-abdominal infection = 12; urinary tract infection = 4; and sinusitis = 1)]. Thus, 76 patients with bacteremia/complicated MRSA infections were analyzed for treatment efficacy (53 in the high loading dose regimen group and 23 in the enhanced high loading dose regimen group). Teicoplanin MICs were ≤ 2 μg/mL in all MRSA isolates, and there was no resistant strain. Baseline demographics of enrolled patients with the conventional and enhanced high loading dose regimens are shown in Table 2. The total doses for the initial 3 days and the maintenance dose after day 4 according to renal function in patients with conventional and enhanced high loading dose regimens are shown in supplemental Table 1. The recommended doses and observed administered doses in this study were similar in each renal function category.

**Table 2:** Baseline demographics of patients included in the pharmacokinetics, safety, and clinical efficacy analyses

Baseline demographics	All patients (*n* = 512)			Patients in clinical efficacy population (*n* = 76)		
	Conventional high loading dose regimen (*n* = 363)	Enhanced high loading dose regimen (*n* = 149)	*P* value	Conventional high loading dose regimen (*n* = 53)	Enhanced high loading dose regimen (*n* = 23)	*P* value
Sex male (%)	220 (60.6%)	98 (65.8%)	0.274	33 (62.3%)	19 (82.6%)	0.109
Age (years)	64.9 ± 15.8	67.2 ± 14.2	0.127	69.1 ± 13.1	70.3 ± 14.8	0.737
Body weight (kg)	55.5 ± 11.1	55.7 ± 12.0	0.819	53.0 ± 10.3	55.0 ± 13.8	0.483
Body mass index	21.3 ± 4.0	21.5 ± 4.5	0.787	20.8 ± 3.7	20.2 ± 4.9	0.528
Serum albumin (g/dL)	2.6 ± 0.6	2.5 ± 0.6	0.174	2.5 ± 0.5	2.3 ± 0.5	0.076
Estimated glomerular filtration rate (mL/min/1.73 m2)	69.5 ± 37.7	63.0 ± 33.1	0.065	70.5 ± 38.0	62.7 ± 35.4	0.408
≥60 mL/min/1.73 m2 (Normal renal function)	211 (58.1%)	82 (55.0%)	0.520	33 (62.3%)	13 (56.5%)	0.638
Type of infection						
Bacteremia	107 (29.5%)	48 (32.2%)	0.540	15 (28.3%)	7 (30.4%)	0.851
Infectious endocarditis	3 (0.8%)	4 (2.7%)	0.202	0 (0.0%)	0 (0.0%)	–
Pneumonia (VAP in clinical efficacy population)	59 (16.3%)	21 (14.1%)	0.541	35 (66.0%)	15 (65.2%)	0.945
Osteomyelitis and arthritis	23 (6.3%)	13 (8.7%)	0.337	6 (11.3%)	3 (13.0%)	1.000
Central nervous system infections	0 (0.0%)	1 (0.7%)	0.291	0 (0.0%)	1 (4.3%)	0.303
Intraabdominal infections	61 (16.8%)	24 (16.1%)	0.847	–	–	–
Skin & soft tissue infections	25 (6.9%)	10 (6.7%)	0.943	–	–	–
Urinary tract infections	11 (3.0%)	2 (1.3%)	0.364	–	–	–
Sinusitis	2 (0.6%)	0 (0.0%)	1.000	–	–	–
Mediastinitis	0 (0.0%)	0 (0.0%)	–	–	–	–
Unknown (empiric therapy)	90 (24.8%)	41 (27.5%)	0.521	–	–	–
Isolated Gram-positive organisms						
MRSA	107 (43.9%)	49 (43.8%)	0.986	53 (100%)	23 (100%)	–
MSSA	18 (7.4%)	9 (8.0%)	0.827	–	–	–
MR-CNS	50 (20.5%)	24 (21.4%)	0.840	–	–	–
MS-CNS	7 (2.9%)	1 (0.9%)	0.444	–	–	–
*Enterococcus faecalis*	16 (6.6%)	7 (6.3%)	0.913	–	–	–
*Enterococcus faecium*	41 (16.8%)	23 (20.5%)	0.394	–	–	–
Other Enterococcus sp	10 (4.1%)	0 (0.0%)	0.034	–	–	–
Streptococcus sp	9 (3.7%)	4 (3.6%)	1.000	–	–	–
Gram-positive rod	7 (2.9%)	2 (1.8%)	0.725	–	–	–

Age, body weight, serum albumin and estimated glomerular filtration rate are expressed as the mean ± S.D.

*VAP* ventilator associated pneumonia, *MRSA* methicillin-resistant *Staphylococcus aureus*, *MSSA* methicillin-sensitive *Staphylococcus aureus, MR-CNS* methicillin-resistant coagulase-negative *Staphylococci, MS-CNS* methicillin-sensitive coagulase-negative *Staphylococci*

### Measurements of C_min_

The median C_min_ on the fourth day was 18.3 μg/mL in the conventional high loading dose regimen group, and 24.9 μg/mL in the enhanced high dose loading regimen group (*p* < 0.001) (Table 3). A similar difference was confirmed in each renal function category (Supplementary Table 2). The proportion of patients achieving the target range (20–40 μg/mL) in the enhanced high loading dose regimen was significantly higher than that in the conventional high loading dose regimen (75.2% versus 41.0%, *p* < 0.001). Even in the enhanced high loading dose regimen group, only 5 of 149 patients had a C_min_ ≥ 40 μg/mL and no patient experienced a C_min_ ≥ 60 μg/mL. Additional loading doses were administered if the initial C_min_ was < 20 μg/mL (25 of 32 patients, 78.1%). However, the target C_min_ was ≥15 μg/mL in the conventional high loading dose regimen, and additional loading doses were administered if the initial C_min_ was < 15 μg/mL (56 of 85 patients, 65.9%).

**Table 3:** Teicoplanin initial trough concentration (Cmin) in patients receiving a conventional or enhanced high loading dose regimen

Initial C_min_ (μg/mL)	Conventional high loading dose regimen (*n* = 363)	Enhanced high loading dose regimen (*n* = 149)	*P*-value
Median (interquartile range)	18.3 (15.1–22.8)	24.9 (20.8–28.3)	< 0.001
No. of patient according to the C_min_ categories (%)			
< 20	213 (58.7%)	32 (21.5%)	< 0.001
20–40	149 (41.0%)	112 (75.2%)	< 0.001
≥ 40	1 (0.3%)	5 (3.4%)	0.009

In the multivariate analysis, enhanced high loading dose regimen (adjusted OR: 7.75, 95% CI: 4.62–13.00) and body mass index ≥25 (adjusted OR: 2.33, 95% CI: 1.24–4.38) were independent factors to achieve an initial C_min_ ≥ 20 μg/mL. In contrast, hypoalbuminemia (adjusted OR: 0.24, 95% CI: 0.15–0.37), total parenteral nutrition (adjusted OR: 0.54, 95% CI: 0.32–0.92), and surgery within 28 days (adjusted OR: 0.47, 95% CI: 0.30–0.74) decreased the attainment of an initial C_min_ ≥ 20 μg/mL (Table 4). Although the cut off serum albumin concentration was defined as the median value for hypoalbuminemia, the median C_min_ according to each serum albumin concentration category were 25.7 μg/mL in the ≥3.5 g/dL group; 22.0 μg/mL in the 3.0–3.5 g/dL group; 21.6 μg/mL in the 2.5–3.0 g/dL group; 18.2 μg/mL in the 2.0–2.5 g/dL group; and 16.2 μg/mL in the < 2.0 g/dL group. There was a tendency toward a dose response relationship between C_min_ and serum albumin level.

**Table 4:** Variables associated with a teicoplanin initial trough concentration (C_min_) ≥ 20 μg/mL: univariate and multivariate analyses

Factors	No of patients with teicoplanin initial C_min_ ≥20 μg/mL (%)		Univariate analysis		Multivariate analysis	
	Patients with factor	Patients without factor	Crude odds ratio (95%CI)	*P*-value	Adjusted odds ratio (95%CI)	*P*-value
Enhanced high loading dose regimen	117/149 (78.5%)	150/363 (41.3%)	5.19 (3.33–8.089)	< 0.001	7.75 (4.62–12.99)	< 0.001
Sex (male)	161/318 (50.6%)	106/194 (54.6%)	0.85 (0.60–1.22)	0.378		
Age (> 65 years)	162/318 (50.9%)	105/194 (54.1%)	0.88 (0.62–1.26)	0.485		
Body mass index< 18.5	59/124 (47.6%)	208/388 (53.6%)	0.79 (0.52–1.18)	0.242		
Body mass index ≥25	47/74 (63.5%)	220/438 (50.2%)	1.73 (1.04–2.87)	0.034	2.33 (1.24–4.38)	0.008
eGFR≥60 mL/min/1.73 m^2^	164/293 (56.0%)	103/219 (47.0%)	1.43 (1.01–2.04)	0.045	1.15 (0.68–1.95)	0.603
Heart disease	111/208 (53.4%)	156/304 (51.3%)	1.09 (0.76–1.55)	0.648		
Chronic renal failure	47/116 (40.5%)	220/396 (55.6%)	0.55 (0.36–0.83)	0.004	0.62 (0.38–1.03)	0.062
Diabetes mellitus	58/112 (51.8%)	209/400 (52.3%)	0.98 (0.65–1.49)	0.931		
Collagen disease	25/53 (47.2%)	242/459 (52.7%)	0.80 (0.45–1.42)	0.443		
Chronic respiratory disease	14/27 (51.9%)	253/485 (52.2%)	0.99 (0.46–2.15)	0.975		
Inflammatory bowel disease	44/89 (49.4%)	223/423 (52.7%)	0.88 (0.56–1.39)	0.573		
Intensive care unit stay (> 3 days)	34/98 (34.7%)	233/414 (56.3%)	0.41 (0.26–0.65)	< 0.001	0.32 (0.19–0.56)	< 0.001
Liver cirrhosis/chronic hepatic dysfunction	34/72 (47.2%)	233/440 (53.0%)	0.80 (0.48–1.31)	0.367		
Malignant tumor	99/204 (48.5%)	168/308 (54.5%)	0.79 (0.55–1.12)	0.182		
Total parenteral nutrition	45/102 (44.1%)	222/410 (54.1%)	0.70 (0.43–1.03)	0.070	0.54 (0.32–0.92)	0.022
Serum albumin < 2.5 g/dL (median)	79/217 (36.4%)	188/295 (63.7%)	0.33 (0.23–0.47)	< 0.001	0.24 (0.15–0.37)	< 0.001
Ventilator use	37/90 (41.1%)	230/422 (54.5%)	0.58 (0.37–0.93)	0.021	1.51 (0.68–3.36)	0.316
Surgery within 28 days	68/158 (43.0%)	199/354 (56.2%)	0.59 (0.40–0.86)	0.006	0.47 (0.30–0.74)	0.001
Transplantation	6/8 (75.0%)	261/504 (51.8%)	2.79 (0.56–13.97)	0.289		
Steroid use	42/85 (49.4%)	225/427 (52.7%)	0.88 (0.55–1.40)	0.580		
Immunosuppressive therapy	18/28 (64.3%)	249/484 (51.4%)	1.70 (0.77–3.76)	0.186		
Anticancer therapy	26/47 (55.3%)	241/465 (51.8%)	1.15 (0.63–2.10)	0.648		
MRSA infections	72/139 (51.8%)	195/373 (52.3%)	0.98 (0.66–1.45)	0.923		
Complicated MRSA infections	54/99 (54.5%)	213/413 (51.6%)	1.13 (0.73–1.75)	0.595		
APACHE II score ≥ 15	54/130 (41.5%)	213/382 (55.8%)	0.56 (0.38–0.84)	0.005	0.78 (0.44–1.35)	0.371

*eGFR*, estimated glomerular filtration rate, *APACHE II* Acute physiology and chronic health evaluation II score

### Clinical efficacy of teicoplanin therapy in patients with complicated MRSA infection

Fifty-four of 76 patients (71.1%) met the definition for an early clinical response on the fourth day, and 55 of 76 patients (72.4%) met the definition of clinical success at the end of the therapy. The early clinical response rate in patients with an initial C_min_ ≥ 20 μg/mL tended to be higher than those with a C_min_ < 20 μg/mL [31/39 (79.5%) versus 23/37 (62.2%), *p* = 0.096]. However, there was no significant difference in clinical success at the end of therapy between patients who did and did not achieve an initial C_min_ ≥ 20 μg/mL. The maximum C_min_ during therapy and the type of regimen did not affect any patient outcomes (Table 5, supplementary Table 3). In the multivariate analysis, an initial C_min_ ≥ 20 μg/mL (adjusted OR: 3.95, 95% CI: 1.25–12.53) and bacteremia (adjusted OR: 4.55, 95% CI: 1.10–18.77) were independent factors for an early clinical response to teicoplanin therapy (Table 6).

**Table 5:** Patient outcomes according to the value of the initial and maximal trough concentration (Cmin)

Outcomes	No. of patients with initial C_min_			No. of patients with maximal C_min_		
	< 20 μg/mL	≥20 μg/mL	*P*-value	< 20 μg/mL	≥20 μg/mL	*P*-value
Early clinical response (*n* = 76)	23/37 (62.2%)	31/39 (79.5%)	0.096	–	–	–
Clinical success at the end of therapy (*n* = 76)	25/37 (67.6%)	30/39 (76.9%)	0.362	22/33 (66.7%)	33/43 (76.7%)	0.330
Microbiological success (*n* = 68)	22/33 (66.7%)	25/35 (71.4%)	0.671	20/31 (64.5%)	27/37 (73.0%)	0.452
28 days mortality (*n* = 76)	5/37 (13.5%)	2/39 (5.1%)	0.256	5/33 (15.2%)	2/43 (4.7%)	0.229

Eight patients in whom culture results after the start of therapy were not obtained were excluded from the microbiological success analysis

**Table 6:** Variables associated with the early clinical response of teicoplanin therapy in patients with complicated MRSA infections: univariate and multivariate analyses

Factors	No of patients with early clinical response (%)		Univariate analysis		Multivariate analysis	
	Patients with factor	Patients without factor	Crude odds ratio (95% CI)	*P*-value	Adjusted odds ratio (95%CI)	*P*-value
Enhanced high dose loading regimen	16/23 (69.6%)	38/53 (71.7%)	0.90 (0.31–2.63)	0.851		
Teicoplanin initial C_min_ ≥20 μg/mL	31/39 (79.5%)	23/37 (62.2%)	2.36 (0.09–3.95)	0.096	3.95 (1.25–12.53)	0.020
Blood stream infection	19/22 (86.4%)	33/54 (64.8%)	3.44 (0.90–13.13)	0.060	4.55 (1.10–18.77)	0.036
Respiratory infection	33/50 (66.0%)	21/26 (80.8%)	0.46 (0.15–1.44)	0.178		
Osteomyelitis and arthritis	6/9 (66.7%)	48/67 (71.6%)	0.79 (0.18–3.49)	0.713		
Central nervous system	0/1 (0.0%)	54/75 (72.0%)	–	0.289		
Mixed infection with Gram-negative organisms	22/34 (64.7%)	32/42 (76.2%)	0.57 (0.21–1.58)	0.272		
Therapy for definitive fungal infections	2/2 (100.0%)	52/74 (70.3%)	–	1.000		
Sex (male)	37/52 (71.2%)	17/24 (70.8%)	1.02 (0.35–2.95)	0.977		
Age (> 65 years)	38/57 (66.7%)	16/19 (84.2%)	0.38 (0.10–1.45)	0.144		
eGFR< 30 mL/min/1.73 m^2^	8/13 (61.5%)	46/63 (73.0%)	0.59 (0.17–2.06)	0.504		
Body mass index < 18.5	22/25 (88.0%)	32/51 (62.7%)	4.35 (1.15–16.52)	0.023	2.94 (0.69–12.41)	0.143
Body mass index ≥25	4/6 (66.7%)	50/70 (71.4%)	0.80 (0.14–4.72)	1.000		
Heart disease	29/37 (78.4%)	25/39 (64.1%)	2.03 (0.73–5.63)	0.170		
Chronic renal failure	11/17 (64.7%)	43/59 (72.9%)	0.68 (0.22–2.15)	0.552		
Diabetes mellitus	13/20 (65.0%)	41/56 (73.2%)	0.68 (0.23–2.03)	0.487		
Collagen disease	5/10 (50.0%)	49/66 (74.2%)	0.35 (0.09–1.35)	0.142		
Chronic respiratory disease	4/8 (50.0%)	50/68 (73.5%)	0.36 (0.08–1.59)	0.219		
Inflammatory bowel disease	7/9 (77.8%)	47/67 (70.1%)	1.49 (0.28–7.80)	1.000		
Intensive care unit stay (> 3 days)	13/22 (59.1%)	41/54 (75.9%)	0.46 (0.16–1.31)	0.142		
Liver cirrhosis/chronic hepatic dysfunction	6/12 (50.0%)	48/64 (75.0%)	0.33 (0.09–0.28)	0.094	0.28 (0.07–1.19)	0.084
Malignant tumor	23/33 (69.7%)	31/43 (72.1%)	0.89 (0.33–2.41)	0.819		
Total parenteral nutrition	10/16 (62.5%)	44/60 (73.3%)	0.61 (0.19–1.94)	0.536		
Serum albumin < 2.5 g/dL (median)	32/43 (74.4%)	22/33 (66.7%)	1.46 (0.54–3.94)	0.460		
Ventilator use	11/22 (50.0%)	43/54 (79.6%)	0.26 (0.09–0.74)	0.010	0.54 (0.16–1.86)	0.330
Surgery within 28 days	10/14 (71.4%)	44/62 (71.0%)	1.02 (0.28–3.69)	1.000		
Transplantation	2/3 (66.7%)	52/73 (71.2%)	0.81 (0.07–9.39)	1.000		
Steroid use	9/16 (56.3%)	45/60 (75.0%)	0.43 (0.14–1.35)	0.213		
Immunosuppressive therapy	2/3 (66.7%)	52/73 (71.2%)	0.81 (0.07–9.39)	1.000		
Anticancer therapy	8/12 (66.7%)	46/64 (71.9%)	0.78 (0.21–2.92)	0.736		
APACHE II score ≥ 15	17/28 (60.7%)	37/48 (77.1%)	0.46 (0.17 1.27)	0.129		
Teicoplanin-resistant MRSA (MIC ≥4 μg/mL)	0	54/76 (71.1%)	–	–		

*C*_*min*_ trough concentration, *eGFR* estimated glomerular filtration rate, *APACHE II* Acute physiology and chronic health evaluation II score, *MIC* minimum inhibitory concentration

### Adverse events related to teicoplanin therapy

In the population used for the assessment of safety, there were no significant differences in the occurrence of adverse events on the fourth day and at the end of therapy between those patients who did and did not achieve an C_min_ ≥ 20 μg/mL (nephrotoxicity: 2.9% versus 3.4%, *p* = 0.739, and 7.8% versus 7.9%, respectively; hepatotoxicity: 1.6% versus 1.5%, *p* = 1.000, and 2.9% versus 1.5%, *p* = 0.366, respectively) (Table 7). There was no significant difference in the occurrence of adverse events between the two teicoplanin regimens (supplementary Table 4).

**Table 7:** Adverse effects according to the value of the initial and maximal trough concentration (Cmin)

Adverse effects	No. of patients with initial C_min_		*P*-value	No. of patients with maximal C_min_		*P*-value
	< 20 μg/mL (*n* = 245)	≥20 μg/mL (*n* = 267)		< 20 μg/mL (*n* = 235)	≥20 μg/mL (*n* = 277)	
Nephrotoxicity on the 4th day	7 (2.9%)	9 (3.4%)	0.739	7 (3.0%)	9 (3.2%)	0.861
Nephrotoxicity at the end of therapy	19 (7.8%)	21 (7.9%)	0.963	17 (7.2%)	23 (8.3%)	0.653
Hepatotoxicity on the 4th day	4 (1.6%)	4 (1.5%)	1.000	4 (1.7%)	4 (1.4%)	1.000
Hepatotoxicity at the end of therapy	7 (2.9%)	4 (1.5%)	0.366	7 (3.0%)	4 (1.4%)	0.360

## Discussion

Although it appears that teicoplanin C_min_ ≥ 15 μg/mL is required for clinical success in the majority of MRSA infections [7, 8]. C_min_ ≥ 20 μg/mL is recommended for serious infections such as infective endocarditis and bone and joint infections. However, the recommendation of this high target C_min_ was based on case-control studies of a small number of patients and statistical analyses were often difficult. To the best of our knowledge, this is the first study to draw the conclusion with the multivariate analyses. Initial C_min_ ≥ 20 μg/mL (adjusted OR: 3.95) was an independent factor for the early clinical response to teicoplanin therapy. However, there was no significant difference in clinical success at the end of therapy between patients who did and did not achieve an initial C_min_ ≥ 20 μg/mL, possibly because of dose modifications based on the initial C_min._

For infective endocarditis and bone and joint infections, teicoplanin 12 mg/kg body weight every 12 h for three to five doses was recommended to achieve a target C_min_ ≥ 20 μg/mL [6]. However, the optimal number of loading doses is unclear. In general, population PK analyses and Monte Carlo simulations are conducted to assess the teicoplanin dosage regimens associated with a high probability of achieving the target C_min_ [13, 14]. In these PK simulation studies, the sample size is small for clinical studies and therefore no conclusions about the clinical implications are possible. Previously, we demonstrated that a C_min_ 15–30 μg/mL was obtained in 68% of patients (mean body weight approximately 50 kg) with a dosing regimen of 600 mg at 12-h intervals for five doses (total dose of 3000 mg) [8]. However, the mean C_min_ remained 20.0 μg/mL, and post-hoc analysis revealed that a target C_min_ ≥ 20 μg/mL was obtained in less than half of the patients.

In a regimen of 12 mg/kg every 12 h for four doses followed by 6 mg/kg once daily, the total dose over 3 days was 54 mg/kg (2700 mg in patients weighing 50 kg), which was less than the total dose of 3000 mg in the regimen using 600 mg for five doses. Therefore, in this study we decided to use 12 mg/kg for five doses in patients with difficult MRSA infections to achieve a target C_min_ ≥ 20 μg/mL. With this enhanced high loading dose regimen, a significantly higher achievement rate of the target C_min_ 20–40 μg/mL was observed compared with the conventional regimen (75.2% versus 41.0%, *p* < 0.001). Even with the enhanced loading dose, only a small number of patients had a C_min_ > 40 μg/mL and no patients experienced a C_min_ > 60 μg/mL, which might cause adverse events related to teicoplanin therapy. Because of the adequate teicoplanin concentration, the enhanced loading dose regimen did not result in a high rate of adverse events compared with the conventional loading dose regimen.

In the multivariate analysis, enhanced regimen and body mass index ≥25 were independent factors associated with a C_min_ ≥ 20 μg/mL. In contrast, hypoalbuminemia, total parenteral nutrition, and surgery were selected as independent factors for the decreased attainment of a C_min_ ≥ 20 μg/mL. Several factors other than dosing regimen affected the teicoplanin concentration. There was significant interpatient variability in teicoplanin PK which complicates the empiric approach to dosing, suggesting the need for TDM. On the basis of a PK study of healthy volunteers, multiple-dose teicoplanin administration from 3 to 12 mg/kg of body weight showed a linear dose-serum concentration relationship [21]. However, the dose-serum concentration in critically ill patients can be highly variable [22–24]. Serum albumin concentrations are an important determinant of PK for antibiotics that have a high binding affinity to albumin such as teicoplanin. Lower albumin concentrations were associated with a higher free (unbound) fraction of antibiotic [25], which increases the distribution and clearance of the drug leading to a reduced total drug concentration [26]. Byrne et al. [14] reported that a low serum albumin concentration was associated with the reduced probability of attaining the target total, but not free, C_min_, which is responsible for antimicrobial activity. Dosing regimens for teicoplanin have been determined according to total C_min_ targets that may not be appropriate for patients with hypoalbuminemia.

There were several limitations in our study. First, this study was conducted retrospectively in a single institution. Second, observer bias should be considered. To limit the bias, a clear rule for clinical success was defined. Third, central catheter-related blood stream infections were included in this study, and a different result may have been obtained for clinical efficacy if only patients with complicated MRSA infections, such as infective endocarditis and bone and joint infections, were analyzed. Fourth, more measurements are required to assess when the target C_min_ was actually achieved in the evaluation of clinical efficacy at the end of therapy. Fifth, plasma concentration time curves were not evaluated to support the data obtained. The AUC is an extremely useful parameter in PK models. In vancomycin, use of AUC determined using a Bayesian approach is recommended to optimize dosing. Lastly, the maintenance dose might be relatively low in our study, which might affect the clinical efficacy at the end of therapy. Lee et al. [27] demonstrated that significantly higher favorable final clinical response rates were found in patients who received a loading dose followed by increased maintenance doses of 6 mg/kg/12 h. than those with standard maintenance doses of 6 mg/kg/24 h.

## Conclusions

In conclusion, a higher target initial C_min_ ≥ 20 μg/mL is likely to be associated with a better early clinical response for the treatment of bacteremia/complicated MRSA infections. Although tree to five doses of teicoplanin 12 mg/kg body weight every 12 h is usually used for bone and joint infections and infective endocarditis, only a regimen of five doses is recommended to reach the optimal C_min_.

## Supplementary information

**Additional file 1: Tables S1–4.** were available as Supplementary data. The availability of data was presented within the additional supporting files. (PPTX 52 kb)

**Additional file 2.**

## Data Availability

The dataset was presented within the additional supporting files.
