# Supplementary material for: Clinical efficacy and safety in patients treated with teicoplanin with a target trough concentration of 20 μg/mL using a regimen of 12 mg/kg for five doses within the initial 3 days
Source: BMC Pharmacol Toxicol. 2020 Jul 8;21:50. doi: 10.1186/s40360-020-00424-3 (PMC7341468; doi:10.1186/s40360-020-00424-3)
Supplement: Supplementary file 1 — Additional file 1: Tables S1–4. were available as Supplementary data. The availability of data was presented within the additional supporting files. (PPTX 52 kb) [file 40360_2020_424_MOESM1_ESM.pptx]

## Slide 1
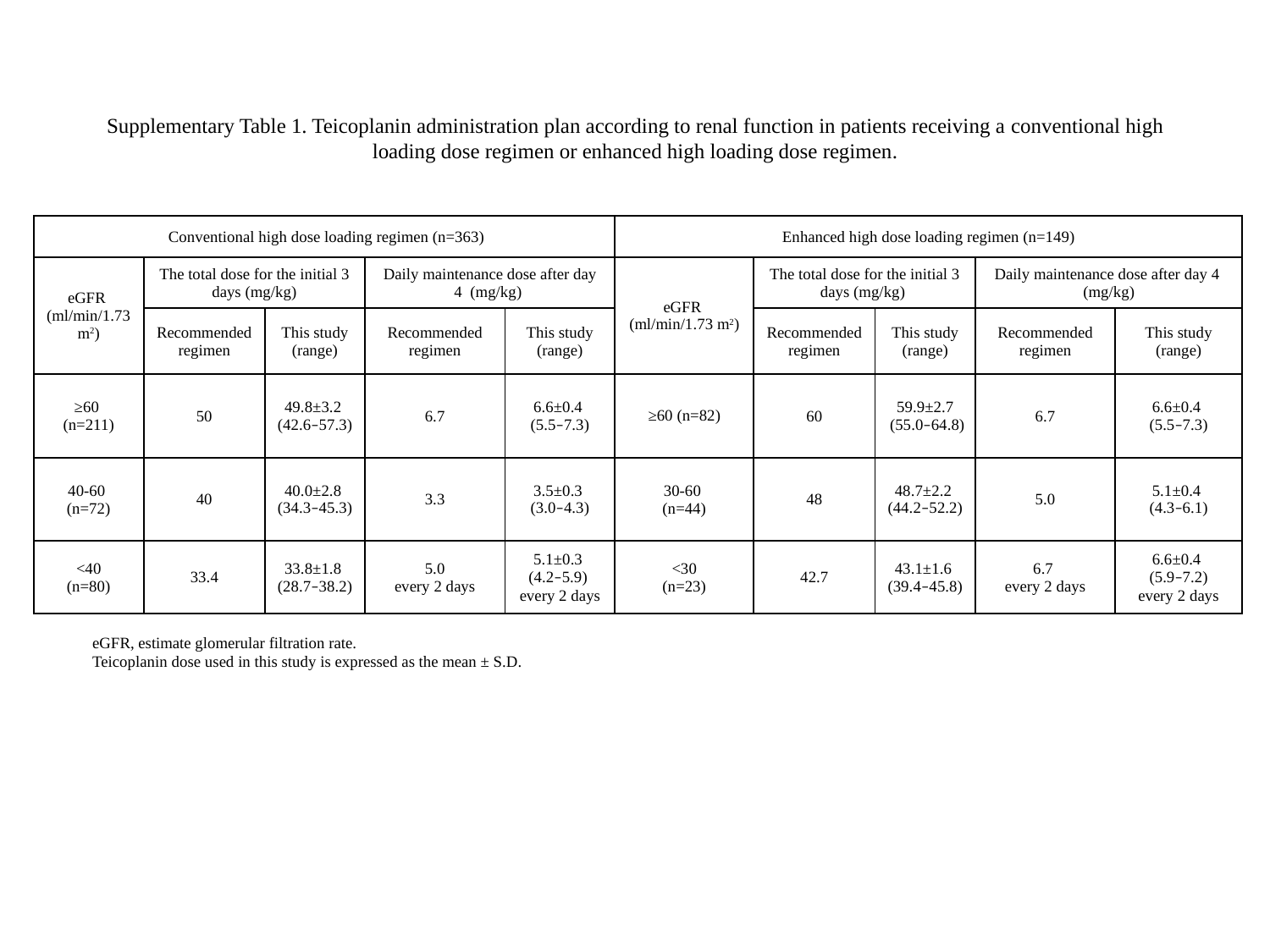

# Supplementary Table 1. Teicoplanin administration plan according to renal function in patients receiving a conventional high loading dose regimen or enhanced high loading dose regimen.
| Conventional high dose loading regimen (n=363) | | | | | Enhanced high dose loading regimen (n=149) | | | | |
| --- | --- | --- | --- | --- | --- | --- | --- | --- | --- |
| eGFR (ml/min/1.73 m2) | The total dose for the initial 3 days (mg/kg) | | Daily maintenance dose after day 4 (mg/kg) | | eGFR (ml/min/1.73 m2) | The total dose for the initial 3 days (mg/kg) | | Daily maintenance dose after day 4 (mg/kg) | |
| | Recommended regimen | This study (range) | Recommended regimen | This study (range) | | Recommended regimen | This study (range) | Recommended regimen | This study (range) |
| ≥60 (n=211) | 50 | 49.8±3.2 (42.6–57.3) | 6.7 | 6.6±0.4 (5.5–7.3) | ≥60 (n=82) | 60 | 59.9±2.7 (55.0–64.8) | 6.7 | 6.6±0.4 (5.5–7.3) |
| 40-60 (n=72) | 40 | 40.0±2.8 (34.3–45.3) | 3.3 | 3.5±0.3 (3.0–4.3) | 30-60 (n=44) | 48 | 48.7±2.2 (44.2–52.2) | 5.0 | 5.1±0.4 (4.3–6.1) |
| <40 (n=80) | 33.4 | 33.8±1.8 (28.7–38.2) | 5.0 every 2 days | 5.1±0.3 (4.2–5.9) every 2 days | <30 (n=23) | 42.7 | 43.1±1.6 (39.4–45.8) | 6.7 every 2 days | 6.6±0.4 (5.9–7.2) every 2 days |
eGFR, estimate glomerular filtration rate.
Teicoplanin dose used in this study is expressed as the mean ± S.D.

## Slide 2
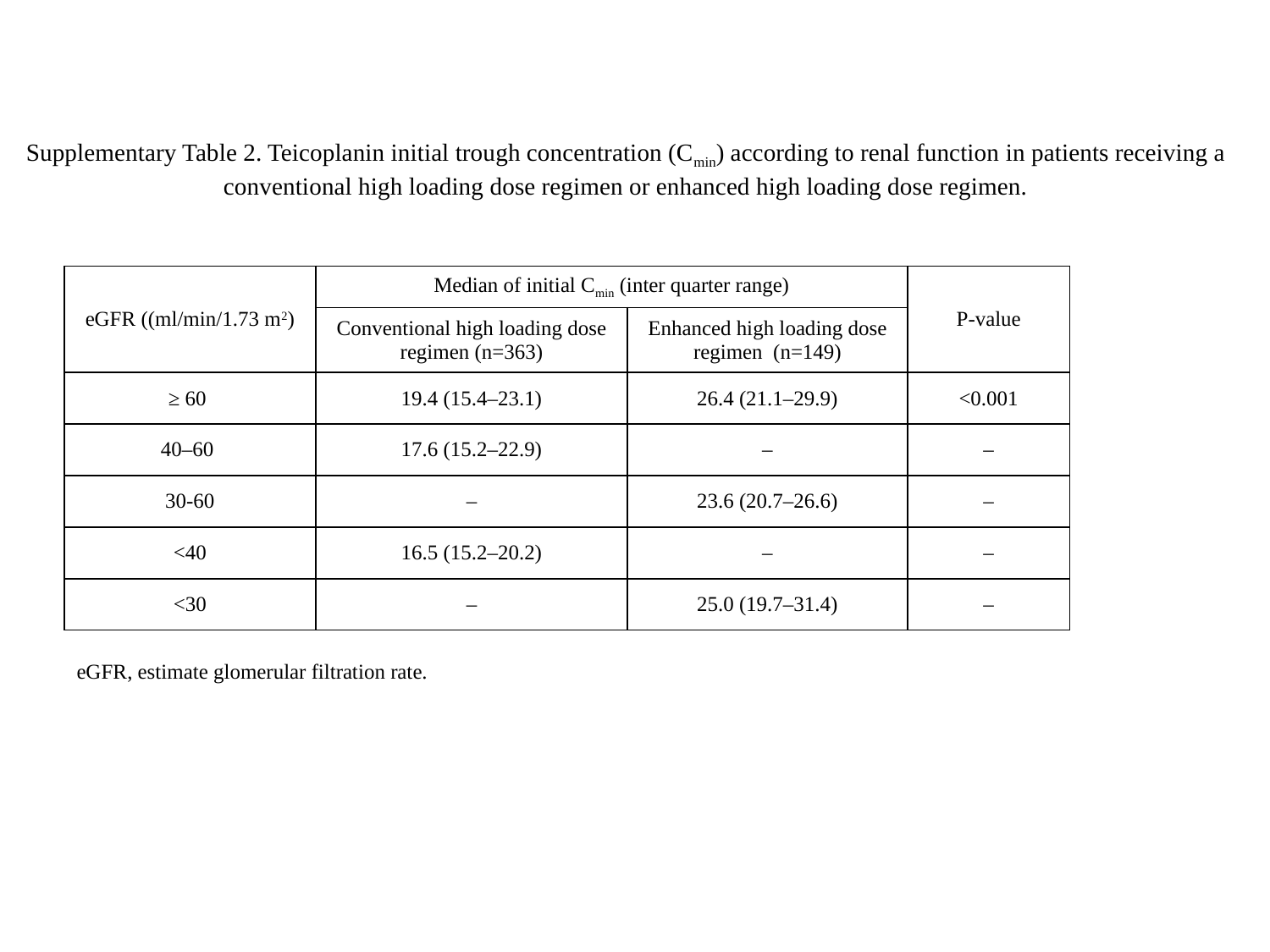

Supplementary Table 2. Teicoplanin initial trough concentration (Cmin) according to renal function in patients receiving a conventional high loading dose regimen or enhanced high loading dose regimen.
| eGFR ((ml/min/1.73 m2) | Median of initial Cmin (inter quarter range) | | P-value |
| --- | --- | --- | --- |
| | Conventional high loading dose regimen (n=363) | Enhanced high loading dose regimen (n=149) | |
| ≥ 60 | 19.4 (15.4–23.1) | 26.4 (21.1–29.9) | <0.001 |
| 40–60 | 17.6 (15.2–22.9) | – | – |
| 30-60 | – | 23.6 (20.7–26.6) | – |
| <40 | 16.5 (15.2–20.2) | – | – |
| <30 | – | 25.0 (19.7–31.4) | – |
eGFR, estimate glomerular filtration rate.

## Slide 3
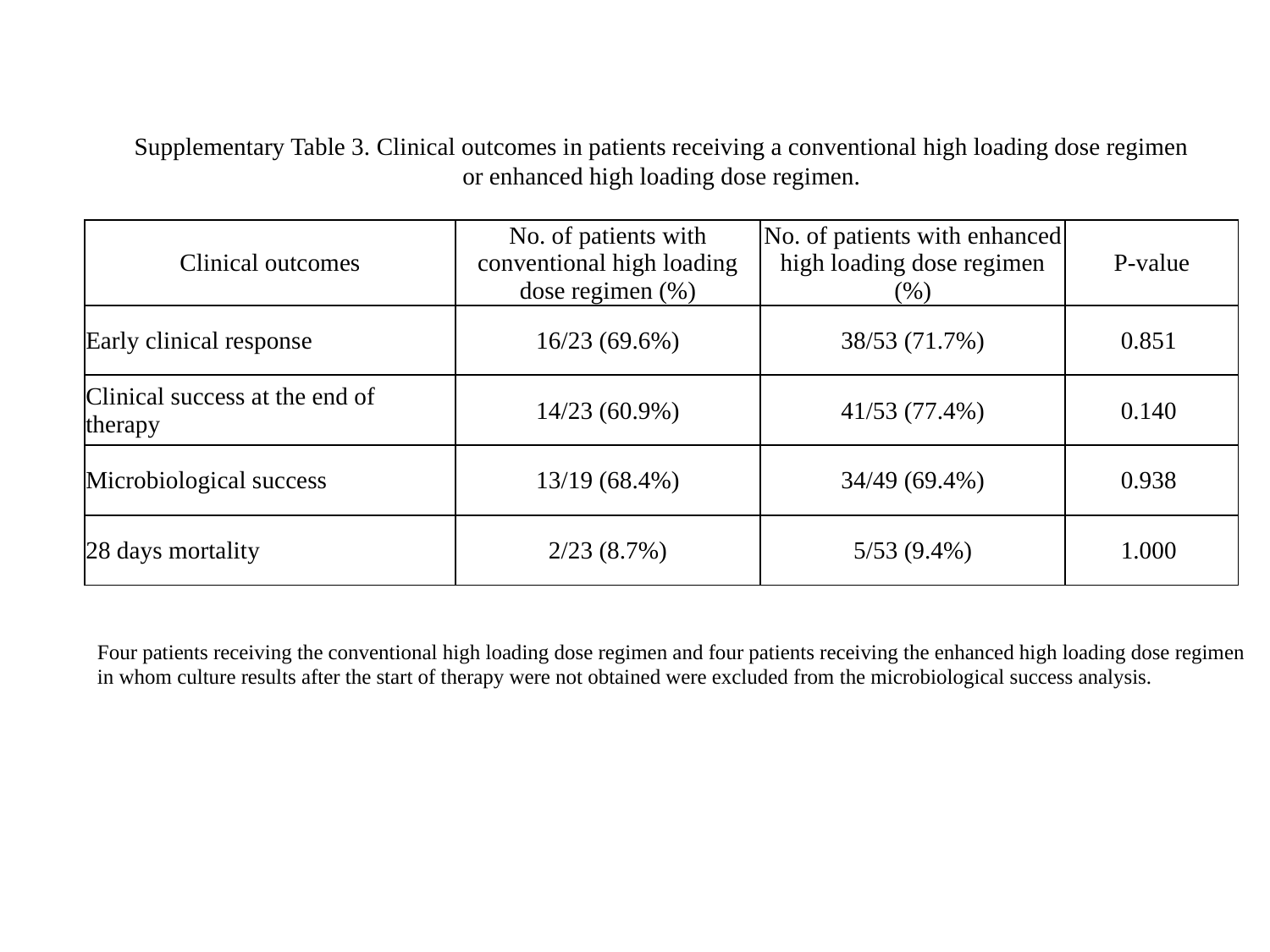

Supplementary Table 3. Clinical outcomes in patients receiving a conventional high loading dose regimen or enhanced high loading dose regimen.
| Clinical outcomes | No. of patients with conventional high loading dose regimen (%) | No. of patients with enhanced high loading dose regimen (%) | P-value |
| --- | --- | --- | --- |
| Early clinical response | 16/23 (69.6%) | 38/53 (71.7%) | 0.851 |
| Clinical success at the end of therapy | 14/23 (60.9%) | 41/53 (77.4%) | 0.140 |
| Microbiological success | 13/19 (68.4%) | 34/49 (69.4%) | 0.938 |
| 28 days mortality | 2/23 (8.7%) | 5/53 (9.4%) | 1.000 |
Four patients receiving the conventional high loading dose regimen and four patients receiving the enhanced high loading dose regimen in whom culture results after the start of therapy were not obtained were excluded from the microbiological success analysis.

## Slide 4
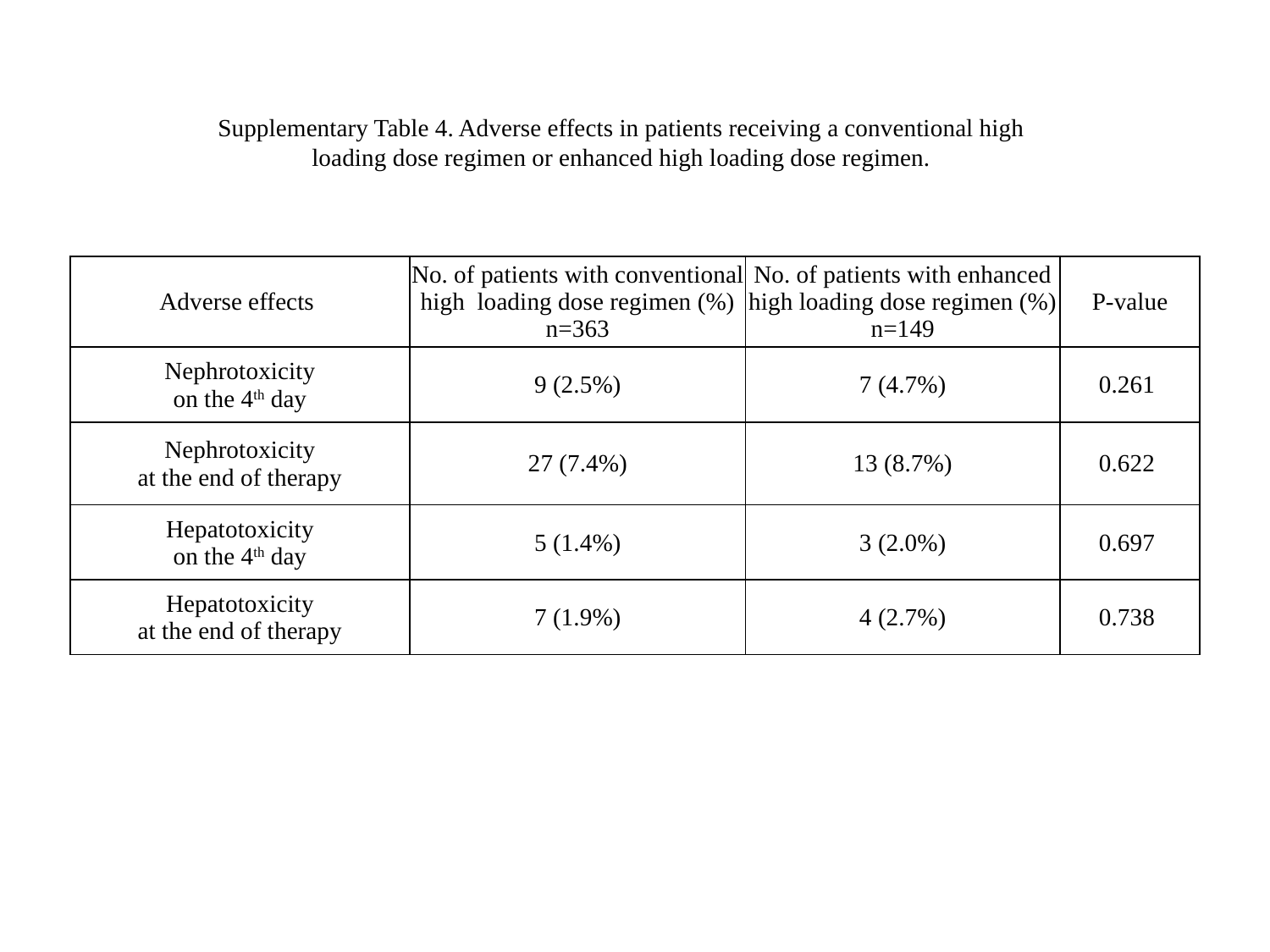

Supplementary Table 4. Adverse effects in patients receiving a conventional high loading dose regimen or enhanced high loading dose regimen.
| Adverse effects | No. of patients with conventional high loading dose regimen (%) n=363 | No. of patients with enhanced high loading dose regimen (%) n=149 | P-value |
| --- | --- | --- | --- |
| Nephrotoxicity on the 4th day | 9 (2.5%) | 7 (4.7%) | 0.261 |
| Nephrotoxicity at the end of therapy | 27 (7.4%) | 13 (8.7%) | 0.622 |
| Hepatotoxicity on the 4th day | 5 (1.4%) | 3 (2.0%) | 0.697 |
| Hepatotoxicity at the end of therapy | 7 (1.9%) | 4 (2.7%) | 0.738 |
